# New Generation of Meso and Antiprogestins (SPRMs) into the Osteoporosis Approach

**DOI:** 10.3390/molecules26216491

**Published:** 2021-10-27

**Authors:** Magdalena Woźniczka, Katarzyna Błaszczak-Świątkiewicz

**Affiliations:** Department of Physical and Biocoordination Chemistry, Faculty of Pharmacy, Medical University of Lodz, Muszyńskiego 1, 90-151 Lodz, Poland; magdalena.wozniczka@umed.lodz.pl

**Keywords:** RANK-RANKL system, antiprogestin, mesoprogestin, osteoporosis, hormone-dependent diseases

## Abstract

Receptor activator of nuclear factor κB (RANK) and its ligand (RANKL) play key roles in bone metabolism and the immune system. The RANK/RANKL complex has also been shown to be critical in the formation of mammary epithelia cells. The female hormones estradiol and progesterone closely control the action of RANKL with RANK. Blood concentration of these sex hormones in the postmenopausal period leads to an increase in RANK/RANKL signaling and are a major cause of women’s osteoporosis, characterized by altered bone mineralization. Knowledge of the biochemical relationships between hormones and RANK/RANKL signaling provides the opportunity to design novel therapeutic agents to inhibit bone loss, based on the anti-RANKL treatment and inhibition of its interaction with the RANK receptor. The new generation of both anti- and mesoprogestins that inhibit the NF-κB-cyclin D1 axis and blocks the binding of RANKL to RANK can be considered as a potential source of new RANK receptor ligands with anti-RANKL function, which may provide a new perspective into osteoporosis treatment itself as well as limit the osteoporosis rise during breast cancer metastasis to the bone.

## 1. Introduction

### 1.1. Molecular and Functional Characterization of RANK and RANKL

TNF (the tumor necrosis factor) and TNFR (the tumor necrosis factor receptor), as well as many related proteins, especially FAS, CD40, CD27, and RANK [1,2], have structural features that are directly linked to the signaling pathways of cell proliferation, survival, and differentiation. TNF/TNFR targets strategies are a subject of research to combat many popular diseases, such as atherosclerosis, allograft rejection, arthritis, and some cancers. Above all members of this large family, RANKL and its RANK receptor are major targets in the development of new therapies for bone-metabolism diseases, such as osteoporosis, tooth loss, arthritis, or cancer bone metastases [3]. RANKL plays an essential role in the expansion of osteolytic metastasis in bones. It is highly expressed in the osteolytic lesions associated with malignant tumors [4]. Osteolytic lesions have been successfully stopped in several types of cancers, including multiple myeloma (MM) and prostate cancer in the way of RANK/RANKL pathway signaling inhibition [5,6].

RANKL (receptor activator of NF-κB ligand, also known as osteoprotegerin-ligand OPG-L, TRANCE, ODF, and TNRSF11), expressed by stromal cells/osteoblasts, is a type II transmembrane protein with an extracellular domain at the carboxy–terminus [3,7]. This ligand has been identified as a factor that can generate osteoclast differentiation and their activation and plays a crucial role in bone physiology. RANKL as a homotrimer interacts with its receptor RANK [7,8]. The RANKL/RANK system leads to osteoclastogenesis from progenitor cells and influences the activation of mature osteoclasts [3]. RANKL is formed in the membrane, and then, after proteolytic cleavage by metalloproteinase enzymes, it can be released into the extracellular environment as a soluble molecule. Both membrane and soluble proteins react with RANK, but binding with membrane-bound RANKL is more effective than with the soluble form [7]. RANK (receptor activator of NF-κB, also known as TRANCE-R, or TNFRSF11A) is a transmembrane heterotrimer. It occurs on the surface of progenitor and mature osteoclasts and mammary gland epithelial cells [3]. The RANKL/RANK molecular pathway is activated by TNF receptor-associated factor (TRAF) so as to induce the differentiation of osteoclast precursors to osteoclasts [9].

### 1.2. The Role of RANK and RANKL in Bone Homeostasis 

The balanced operation between bone forming cells, osteoblasts, and bone resorbing cells, osteoclasts, guarantees the maintenance of bone homeostasis [10]. One of the most important elements for bone remodeling is calcium. Maintaining of the calcium level in the extracellular fluid is done via the bones, which store this element. Calcium is released from bone tissue on the way of RANK and RANKL activation. Parathyroid hormone and calcitonin together with bone participate in the regulation of the calcium concentration in the body. Parathyroid hormone is released as a result of the calcium plasma level reduction. This hormone increases the proliferation of osteoblasts and reduces their apoptosis, which affects the production of calcium [11]. Several bone diseases, such as osteoporosis, rheumatoid arthritis, and periodontitis, have been associated with dysregulation of bone homeostasis by intensive osteoclast formation. Therefore, for therapeutic purpose, it is extremely important to properly modulate the osteoclasts differentiation and activity [10]. 

Osteoclasts are monocyte macrophage lineage multinuclear cells [12] that play an important role in bone metabolism, as well as in other physiological and pathological processes, such as resorption of the ossified callus or inflamed joints [9,13]. Osteoclast differentiation occurs as a result of collective action of macrophage-colony stimulating factor (M-CSF) and RANKL bounded to RANK [10]. Therefore, the balance between osteoclasts and osteoblasts is regulated and RANKL/RANK signal transduction is of upmost importance for the molecular pathway in this process [9,14]. Moreover, it is known that the hypoxia-inducible factor (HIF-2α) plays a significant role in bone remodeling as a regulator of the osteoblast–osteoclast interaction by directly increasing RANKL expression in preosteoblasts. Thus, HIF-2α is also a main controller in the maintenance of bone homeostasis [15]. 

### 1.3. Effect of RANK/RANKL Inhibition in Osteoclastogenesis

To inhibit osteoclastogenesis, the production of osteoprotegerin (OPG, “protector of the bone”, also called the osteoclastogenesis inhibitory factor, OCIF) should be induced. The OPG homodimer, a member of the TNFR family, acts as a soluble decoy receptor for RANKL and competes with RANK for the binding site with RANKL. This makes OPG the effective inhibitor of osteoclast maturation and activation; therefore, OPG is called the osteoclast-mediated bone resorption inhibitor [3]. The balance between RANK/RANKL signaling and the level of OPG regulate the development and activation of osteoclasts and bone metabolism. Most of the factors that regulate OPG expression also induce RANKL expression in osteoblasts. In general, when RANKL expression is up-regulated, expression of OPG is down-regulated. Thus, the RANKL/OPG ratio is changed in favor of osteoclastogenesis [16,17]. In addition, the OPG/RANK/RANKL signaling pathway may play an important role in both physiological and pathological calcification processes, as has been observed in the osteoporotic patient population with the high percentage of vascular calcification incidences [3]. Additionally, it has been demonstrated that alterations in the OPG/RANKL ratio may occur with estrogen deficiency, hyperparathyroidism, and other disorders that stimulate bone resorption what constitutes proof of RANK/RANKL hormone-dependent mediated processes [18,19]. The expression of RANKL and OPG is strongly associated with changes in the levels of pro-inflammatory cytokines as well as hormones such as vitamin D3, PTH (parathyroid hormone), female sex hormones such as progesterone and estrogen, and hormones involved in lactation, e.g., prolactin and parathyroid hormone peptide (PTHrP) [16,20,21]. The major physiological effect of estrogen action is the inhibition of bone resorption [22]. Low estrogen levels are closely associated with the development of postmenopausal osteoporosis. Exposure to 17b-estradiol, the estrogen agonist, improves osteoblastic cell viability and up-regulates the level of OPG [23]. Estradiol induces OPG expression through estrogen receptor (ER) at both mRNA and protein levels. However, no effect on RANKL expression was observed and the estrogen receptor inhibitor did not attenuate estradiol-induced OPG expression [24,25].

The in vitro effects suggest that the relative concentration of calcitonin gene-related peptide (CGRP) in bone may be an important determinant of bone mass and strength [26]. This hypothesis was supported by the analysis which showed that αCGRP−/− mice exhibited an osteopenic phenotype due to a reduction of bone formation [27]. CGRP has been shown to inhibit vitamin D3-stimulated osteoclastogenesis and bone resorption [26,28,29]. Moreover, using the NF-κB-luciferase reporter transgenic mice, CGRP selectively inhibited NF-κB-mediated transcription in thymocytes [30]. This suggests that the inhibitory effect of CGRP on osteoclast formation may be attributed to an inhibitory effect of NF-κB activation in osteoclast precursors [26].

RANK-Fc (RANK coupled with the Fc domain of the human immunoglobulin G1 (IgG1)) has been discovered as a molecule with the ability to react with RANKL in a manner similar to that of OPG. RANK-Fc inhibits bone resorption and prevents tumor-induced hypercalcemia [31,32]. It is a soluble fusion protein that consists of the extracellular domain of RANK coupled with the Fc domain of the human immunoglobulin G1 (IgG1), which generally results in functional inhibition of osteoclastogenesis [5]. Osteosarcoma (OS) is the most popular primary malignant bone tumor, and mainly affects adolescents and young adults. The reports indicate that the RANK-RANKL axis could be manipulated to inhibit OS cells, and provide an access for possible therapy. In vivo, a tumor model evaluating the purely effects on OS cell metastasis revealed that RANK-Fc was effective against tumor spread from the orthotopic site to the lungs, a common site of metastasis in human OS. The treatment with RANK-Fc as a RANKL-targeting agent leads to direct inhibition of OS cell tumorigenicity in vitro and an accompanying inhibition of tumor growth and development of metastases in vivo. Furthermore, the RANK-Fc arresting effects are stronger than the RANKL supportive effects on OS cells [33]. While the other antagonist of RANK, such as OPG or Fc-OPG, can induce an immunological answer [34], RANK-Fc does not rise the host immune response and cannot disturb the TRAIL (TNF-related apoptosis-inducing ligand) pathway [32]. It has been proven that RANK-Fc has a potential advantage over OPG, with greater specificity for RANKL. This may be significant, because it has been shown that multiple myeloma undergoes apoptosis in response to TRAIL. Fusion of RANK to the constant region of hIgG1 dictates homodimerization, which probably increases its avidity for RANKL. RANK-Fc blocks multiple myeloma-induced osteoclastogenesis. Once it is bound by RANK-Fc, RANKL is unable to bind to cell-expressed RANK [6].

The phenomenon of RANK-Fc discovery has allowed the launch of a new monoclonal antibody for RANKL-Denosumab on the market, which successfully inhibits bone resorption [35,36,37]. Denosumab is characterized by long half-life circulation and a lasting effect of the serum level reduction of the resorption and stimulation of bone formation markers. Nevertheless, it will be important to determine in the phase IV clinical trial if long-term inhibition of RANKL causes undesirable side effects in these tissues and the immune response [16].

## 2. Prevention of Osteoporosis—Women’s Health

### 2.1. Hormone-Dependent Diseases with Bone Density Loss

Deterioration of ovarian function and low estrogen serum level in postmenopausal women lead to postmenopausal osteoporosis [38]. In the course of osteoporosis, bone mass decreases and there is a micro-architectonic degradation of bone tissue, which carries the risk of fractures [39,40]. Estrogen, which inhibits the formation of osteoclasts, has a positive effect on osteoporosis inhibition [41]. Moreover, estrogen blocks osteoblast apoptosis and increases their viability by Sema3A secretion induction in osteocytes [42,43]. Sema3A is a membrane-associated secreted protein involved either in bone resorption and its formation.

Estradiol and progesterone strongly control RANKL and RANK complex function [44]. Directly, estradiol can affect bone processes through the inhibition of the RANK+ cells expression, particularly RANK+ monocytes, observed in bone marrow (inter alia by the up-regulation of OPG production in osteoblasts). Estrogen has been shown to down-regulate RANKL production in lymphocytes and modulate production of inflammatory cytokines, which eventually cause bone resorption in osteoporosis as well as rheumatoid arthritis [45]. Based on the in vivo studies in bone resorption approach conducted so far, a number of proinflammatory cytokines have been recognized as mediators of this stage, including interleukin (IL)-1β, IL-6, IL-7. It is known that treatment with IL-1 receptor antagonist or TNF-binding protein decreases osteoclast formation and bone resorption in ovariectomized mice, which indicates that both factors are highly relevant for estrogen-related bone loss [46]. Effects of estrogen on TNF level is indirectly meditated by T cells, revealing the importance of T cells for estrogen-mediated bone protection [41,42,47,48,49].

The effect of 17β-estradiol (ED) on the in vivo and ex vivo differentiation of bone marrow (BM) cells in a mouse collagenase-induced osteoarthritis model (CIOA) indicates a potential preservation role of estradiol against the progress of osteoarthritis (OA). The disease is not only characterized by cartilage degradation, but also involves subchondral bone remodeling that may lead to osteophyte formation at the margin of articular surface. Estradiol treatment diminished cartilage destruction and mainly suppressed osteophyte formation. The results showed that in CIOA mice model, committed monocytoid RANK+pre-OCs were represented in the marrow, forming a pool of cells capable to respond rapidly to RANKL expression. Estradiol treatment inhibits this effect that can result in a decrease of osteoclast formation. Estrogen can inhibit the generation of osteoclast precursors before they are released into circulation. The development of CIOA leads to an increase of cells expressing RANK, and applying 17β-estradiol after the active phase of arthritis, when osteoclast formation is severe, does not compensate this process. 

The RANK/RANKL pathway has also been found to be important in osteoclast-independent mechanisms, mainly in mammary physiology and breast cancer, affecting the proliferation of epithelial cells and cellular survival [50]. It appears that the RANK/RANKL signaling pathway is involved in all stages of the development of breast cancer, from the expansion of the partition and enhancement of the proliferation of epithelial cells to increasing the resistance of tumor cells in anticancer therapy as well as the promotion of damaging agents and metastatic potential [51]. Breast cancer and osteoporosis are common among postmenopausal women. There are existent biochemical links between these two disorders, which might rally around the cellular and molecular pathways based on the RANK actions and its inactions. Increasing the RANKL level encourages osteoclast differentiation with subsequent induction of excessive bone resorption, which results in loss of bone integrity [52].

**Figure 1:**
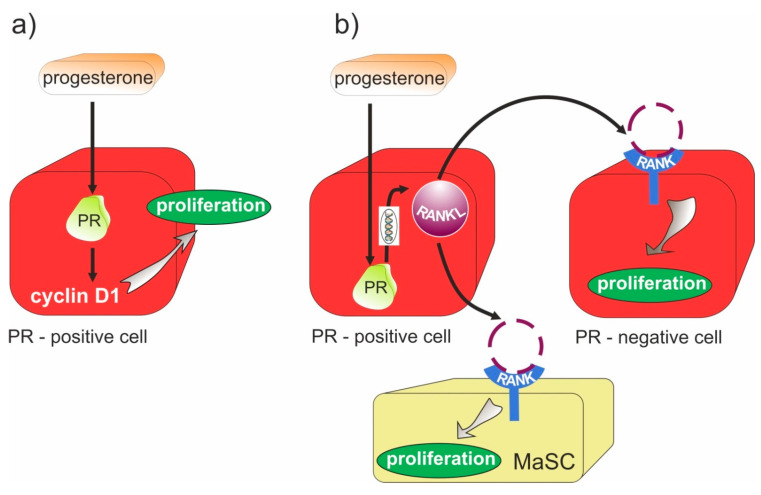
Progesterone (P4)/progesterone receptor (PR) signaling and RANK/RANKL pathway in cells proliferation. (**a**) autocrine mechanism mediated by the activation of cyclin D1 in cells. (**b**) Paracrine cell proliferation. Up-regulation of RANKL by P4 action in PR-positive cells causes RANK/RANKL complex expression in PR-negative cells; therefore, the RANK-NF-κB proliferative pathway is stimulated. Moreover, RANK/RANKL expression in mammary stem cells (MaSCs) activates their expansion.

One of the clinically important molecular effects involves the interaction of the progesterone signaling mechanisms and the RANK/RANKL pathway [50,53,54]. There is a direct effect of progesterone at the progesterone receptor (PR)-expressing cells through cyclin D1-dependent interactions (Figure 1). Cyclin D1 acts as a mitogenic sensor and integrates extracellular mitogenic signals and cell cycle progression. When deregulated (overexpressed, accumulated, and inappropriately located), cyclin D1 becomes an oncogene that is recognized as a driver of solid tumors and hemopathies [55]. The progesterone/progesterone receptor inhibitors block nuclear factor κB activation. The ability of progesterone to inhibit NF-κB expression is associated with rapid induction of the protein level, which blocks NF-κB transactivation [56]. NF-κ, as a well-known molecule, is involved in various metastatic bone diseases. NF-κB is associated with osteoporosis, with a predominant role in osteoclastogenesis. Additionally, the NF-κB signaling pathway is an important mediator in osteoblast differentiation. NF-κB acts as a negative regulator in osteoblast differentiation, which regulates the process of osteoporosis. Therefore, constitutive inhibition of NF-κB promotes osteoblast differentiation what explains positive action against osteoporosis [57].

The regulatory activity of RANKL is an important factor in progesterone-induced breast proliferation. Both RANKL and progesterone are present in the luminal epithelial cells, with increasing progesterone level and RANKL in PR-positive cells (Figure 1). In turn, RANK induces an epithelial-mesenchymal transition and promotes tumor formation and metastasis. As a result of the RANKL-induced paracrine effect, proliferation of PR-negative breast epithelial cells occurs [50]. Experiments in human mammary epithelial cells taken either from the primary breast cancer and the contralateral breast showed high RANKL expression in receptor positive breast cancers. In normal breast and in breast cancer, mRNA for RANKL and RANKL protein expression fluctuate together with serum progesterone fluctuation with the highest level in the luteal phase, suggesting that RANKL is a progesterone signaling modulator in normal and malignant breast tissue and a potential biomarker of progesterone activity and its blockade [58]. Inhibition of the RANK/RANKL pathway could, therefore, serve as a potential target for the prevention and treatment of breast cancer [50].

Long-term immunosuppression or intensive hormone therapy is not a preferable therapy for its excessive and unfathomable consequences for human physiology [42]. While an individual woman may benefit from hormone replacement therapy (HRT) to control menopausal symptoms, it is well known that using combination hormones with both estrogen and progesterone is associated with an increased risk of breast cancer. This risk increases with the duration of HRT and is also higher in women who start HRT close to the onset of menopause rather than later. If estrogens are used alone, most studies do not indicate an increased risk of breast cancer [59]. Therefore, a very specific stimulation of immune-related ERα/β could be a solution. Unraveling conjoined mechanisms of the immune system and bone offer therapeutic possibilities for ailments of both systems [42].

The detection of the estrogen receptor (ER), progesterone receptor (PR), and human epidermal growth factor receptor (HER2) assist the determination of prognosis and treatment choice of breast cancer for clinical practice [60]. The ER/PR balance is crucial for clinical and therapeutic care provided to breast cancer patients. Proper patient classification helps to select hormonal treatment for ER/PR-positive patients and chemotherapy for the ER/PR-negative patients. ERα-negative breast cancer has a poor prognosis partly due to the lack of targeted receptors. The most devastating disease with high morbidity and mortality rate is triple negative breast cancer (TNBC). TNBC is defined by the absence of the three most commonly targeted receptors: ER, PR, and HER2 [61,62].

### 2.2. The Popular Drugs Effective to Osteoporosis Prevention

#### 2.2.1. Denosumab (Prolia, Xgeva) 

Denosumab (XGEVA^®^, Prolia^®^, Amgen Inc., Thousand Oaks, CA, USA) is a fully human monoclonal IgG2 isotype antibody produced in a mammalian cell line (Chinese hamster ovary cells) by recombinant DNA technology. This drug is characterized by its high affinity and specificity to human RANKL, with no detectable binding to TNF-α, TNF-β, TRAIL, or CD40L [31].

Denosumab bounded to RANKL disrupts the RANK–RANKL complex, which is necessary for osteoclast formation; therefore, reduction or elimination of osteoclast is in progress. The denosumab–RANK ligand complex eliminates osteoclastic cells and blocks bone resorption (and bone turnover) in different diseases with bone density loss [31]. In bone caners, consequently, osteolysis is reduced and proliferative tumor stroma is replaced into densely woven new bone [63].

Denosumab, as a native immunoglobulin, is composed solely of amino acids and carbohydrates and its metabolism and elimination follow the immunoglobulin clearance pathways, with degradation to small peptides and individual amino acids [63]. Therefore, no dose adjustments are required for patients with hepatic or renal impairment as well as elderly patients because monoclonal antibodies are not eliminated via hepatic metabolic mechanisms.

The pharmacokinetics of denosumab have been tested for indications of certain diseases, such as osteoporosis and skeletal-related events in patients with bone metastases and certain cancers. Denosumab bioavailability was detected as 61% following subcutaneous administration to patients with advanced solid tumors and 64% in patients with osteoporosis [64,65]. The fundamental pathways for systemic absorption of monoclonal antibodies following subcutaneous administration are connected with the convective transport of the antibody through lymphatic vessels into the blood, and diffusion of the antibody across blood vessels was distributed near the site of injection. Since migration of the lymphatic system is relatively slow, monoclonal antibodies are absorbed over a long period of time after drug administration. In humans, denosumab shows a typical absorption pattern, with a half-life estimated at 79 h (more than 3 days). Clinical trials of the effectiveness of denosumab have been conducted in women with low bone mineral density (BMD) or osteoporosis. Studies have confirmed that denosumab reduced the incidence of new bone fractures and also increased the BMD [65]. Similar results were obtained in men receiving androgen deprivation therapy for non-metastatic prostate cancer [66].

Phase I and II clinical trials results have shown that denosumab suppresses bone resorption in patients with malignant bone disease with multiple myeloma or breast cancer [67]. Clinical studies have reported that denosumab suppresses osteolytic activity and slows down the progression of bone tumor giant cells (GCTB). GCTB is characterized by neoplastic stromal cells that expressed RANKL and osteoclast-like giant cells with RANK expression. RANKL appears to be a main element in the pathogenesis of GCTB [67,68,69].

Potentially, denosumab may be used for the prophylactic treatment of breast cancer in BRCA1/2 genetically predisposed patients. BRCA1 and BRCA2 mutations are the most prevalent genetic drivers for hereditary breast cancer in humans. Healthy women with a BRCA1 mutation will be able to benefit from denosumab treatment. In a pilot clinical study, the proliferation marker Ki67 was significantly down-regulated in the breast biopsy of BRCA1 mutation carriers who received short-term treatment with denosumab, suggesting that RANKL inhibition appears as a practicable method for the chemoprevention of breast cancer in women with BRCA1 mutations [70,71].

#### 2.2.2. Bisphosphonates (Fosamax, Bonviva, Aclasta, and Zometa)

Bisphosphonates (BPNs) are a class of osteoclasts and bone resorption inhibitors [72]. The potent embedding of bisphosphonates to bone has been recognized as a unique property of bisphosphonates that gives them selectivity to their intended target organ [73]. Based on their two phosphonate groups, bisphosphonates selectively bind to bone mineral and act as osteoclasts inactivators, the cells that break down bone tissue, thereby functioning as bone resorption inhibitors. Although both bisphosphonates and denosumab reduce the osteoclast activity, their action mechanism is totally different. Bisphosphonates bind to the bone mineral, prevent the inhibitory effect of mature osteoclasts, while denosumab precludes the binding of RANKL to its receptor RANK [74,75]. The process of bisphosphonate action does not appear to be as powerful as compared to the RANKL inhibition, since osteoclasts are not cleared after exposure to bisphosphonates neither in animals nor humans [73]. The adsorption of bisphosphonates to hydroxyapatite (HAP) bone mineral surfaces brings them into close contact with osteoclasts and other cells in the bone. During bone resorption, the acidic environment created by the osteoclast in the resorption lacuna is thought to allow the dissociation of bisphosphonates from HAP, since the ability of bisphosphonates to bind to HAP decreases when pH goes down. The bisphosphonate molecule—perhaps complexed with calcium and bone organic matrix proteins—is then taken up by the osteoclast. The strong affinity of bisphosphonates to HAP and bone mineral may limit their distribution throughout the skeleton, particularly deeply to sites in bones. However, bisphosphonates with lower affinity to bone mineral appear to be able to penetrate deeper into the network [73,76].

**Figure 2:**
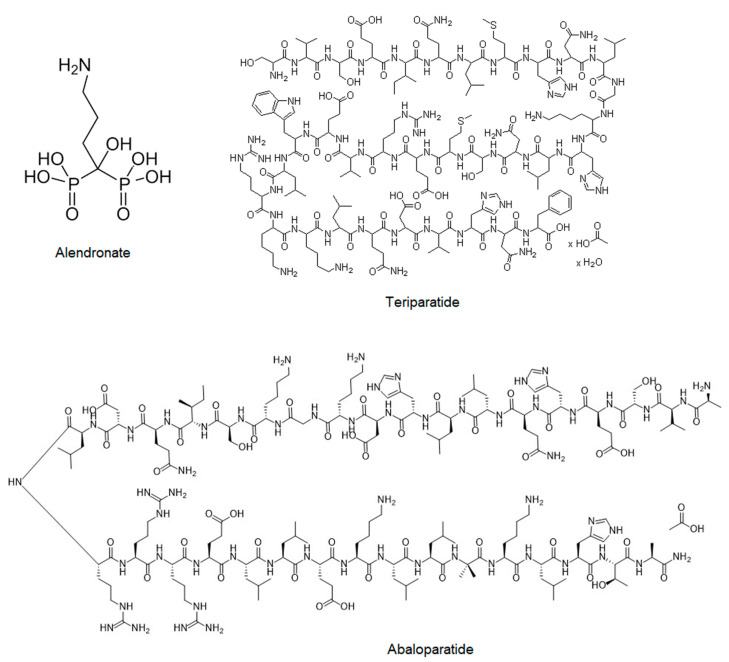
Structure of drugs used in the prevention of osteoporosis.

There are two different types of bisphosphonates, nitrogenous and non-nitrogenous. Nitrogen-bisphosphonates, such as alendronate (Figure 2), ibandronate, minodronate, pamidronate, risedronate, neridronate. and zoledronate, have a side chain that contains a nitrogen atom, whereas the non-nitrogen-bisphosphonates, such as clodronate, tiludronate, and etidronate, do not [73]. Nitrogenous bisphosphonates disrupt osteoclast formation, survival, and cytoskeletal dynamics. Nitrogen-bisphosphonates inhibit the action of enzymes in the mevalonate-cholesterol pathway, thereby contributing to macrophage apoptosis. Non-nitrogenous bisphosphonates affect the apoptosis of osteoclasts. Unfortunately, it is difficult to compare bisphosphonates, as they show different efficacies and oral absorptions [77,78].

Bisphosphonates used in post-menopausal women to treat osteoporosis increase bone density, decrease bone turnover, and reduce fractures. During bisphosphonate therapy in post-menopausal women with osteoporosis increased BMD decreases fracture rates [79]. Nevertheless, caution is advised, because improving bone density without altering resiliency may not lead to the desired life comfort improvements. A report of bisphosphonate-induced osteopetrosis validated these concerns [78]. Bisphosphonates as antiresorptive agents reduce the activity of osteoclasts; thus, a reduction in bone resorption appears. Simultaneously, they also reduce the bone remodeling rate, which ultimately may cause slow microcrack repair. As a consequence, these drugs reduce the resorption stage in bone remodeling, and therefore, the bone density, and the long-term bone quality in osteoporotic patients has a chance to be improved. Even though the mineral content of bones increases, the bones become more brittle and damaged. This may provoke local fractures, despite the higher stiffness and strength of the treated bone. The increased rate for the bone volume fraction is higher during therapy with denosumab than when bisphosphonates are applied. Denosumab treatment with doses of 0.1 and 0.3 mg/kg does not increase the damage level in osteoporotic and trabecular bones, while, on the contrary, for cortical bone, these doses may increase the damage level. In the case of bisphosphonates, doses of 0.25 and 0.5 mg do not increase the damage level for osteoporotic bone [74]. It has been reported that among women receiving bisphosphonates with postmenopausal osteoporosis, there have been rare cases of osteonecrosis of the jaw (ONJ) [80], but no causal association has been established. It is known that bisphosphonates intravenous doses cause high initial concentrations of these drugs in the kidney, which is directly correlated with acute renal injury and nephrotoxicity [81].

Osteogenesis imperfecta (OI), otherwise known as brittle bone disease, is one of the most common primary bone fragility disorders in children and adolescents. The mutation in one of the two genes coding collagen I is the main cause of this pathology. Osteogenesis imperfecta is characterized by low bone mass and bone fragility [72]. Unfortunately, treatment of OI patients is limited to supportive care and there is no cure for them. These therapies include orthopedic management and drugs including bisphosphonates. The most useful pharmacologic therapy, treatment with oral and intra-venous (IV) bisphosphonates is routine treatment for OI, since clinical trials of these agents have consistently provided improvements in bone mineral density (BMD) in patients with OI. Bisphosphonates not only increase lumbar spine (LS) BMD, but also improve bone structures, increase mobility, and reduce fracture risk [78,82,83]. In 2019, Bains et al. revealed that among people who were receiving bisphosphonates, BMD and mobility had increased, while fracture rate and scoliosis decreased in comparison with the group who did not take bisphosphonates [72].

Additionally, direct effects of bisphosphonates on monocytes/macrophages may be responsible for the antitumor activity of these drugs. Tumor-associated macrophages (TAMs) have various protumor functions, including promotion of angiogenesis, matrix remodeling (facilitating the invasive process), and suppression of adaptive antitumor immunity [75]. In the patients with multiple myeloma and metastatic solid tumors, bisphosphonates reduce skeleton-related events such as hypercalcemia, pathological fracture, spinal cord compression, bone pain, and surgery or radiation events. This action makes bisphosphonates desirable to use in the management of cancer affecting the bone, in particular multiple myeloma and metastatic solid tumors (for example, metastatic breast, prostate, and lung cancers) [84].

#### 2.2.3. Teriparatide (Forteo)

Human parathyroid hormone PTH is a linear peptide constituted by 84 amino acids that plays a fundamental role in calcium homeostasis. PTH acts as enhancer of Ca resorption in bones and kidneys, respectively. PTH also stimulates production of activated 1,25-dihydroxyvitamin D (1,25(OH)2D), which mobilizes intestinal calcium absorption [85,86]. PTH probably serves the regulation of bone remodeling rather than overall skeletal mass. The biological activity of intact PTH (hPTH 1–84) resides in the N-terminal sequence Parathyroid hormone, depending on the mode of administration, stimulates bone formation and resorption, and can increase or decrease bone mass. Bone formation is stimulated via continuous infusions and daily subcutaneous injections of parathyroid hormone, but have different effects on bone resorption and bone mass. Continuous infusions, which result in a persistent elevation of the serum parathyroid hormone concentration, lead to greater bone resorption than do daily injections. Daily injections of teriparatide over 19 months increased bone mineral density at the lumbar spine and proximal femur and significantly decreased the incidence of vertebral and nonvertebral fractures [87,88].

Teriparatide—a 34-amino acid peptide of hPTH (1–34) (Figure 2)—has been used as a PTH analogue in osteoporosis treatment. The first two amino acids in this peptide structure are obligatory for its biological activity, and it appears that the bone anabolic properties are fully maintained by the foreshortened fragment hPTH(1–31) or its cyclized lactam [89].

Teriparatide has been approved as anabolic agent for osteoporosis therapy to reduce fracture risk in women with postmenopausal osteoporosis [90]. Teriparatide bone formation on otherwise quiescent bone surfaces and bone turnover stimulation has been detected using a classic remodeling cycle involving both osteoclastic resorption and osteoblastic reformation [87,91,92].

In the SHOTZ (the Skeletal Histomorphometry in Patients on Teriparatide or Zoledronic Acid Therapy) study, postmenopausal women with osteoporosis were treated with teriparatide or zoledronic acid. The aim was to investigate the early effects of these two drugs on the quality of the newly formed bone. At 6 months, treatment with teriparatide versus zoledronic acid resulted in lower mineral and higher organic matrix content, increased tissue water content, and lower mineral/matrix, mineral maturity/crystallinity, glycosaminoglycan content, and pyridinoline/divalent enzymatic collagen cross-link ratio. The results suggest that teriparatide and zoledronic acid have differential effects on material properties of newly formed bone at individual remodeling sites, highlighting their different mechanisms of action [90,93].

#### 2.2.4. Abaloparatide (Tymlos)

Abaloparatide (Figure 2) is a novel synthetic 34-amino acid peptide analog of parathyroid hormone-related protein (PTHrP) that was approved in 2017 for postmenopausal osteoporosis as a physiological regulator of bone formation. However, compared to teriparatide, abaloparatide binds to parathyroid hormone 1 receptor (PTH1R) with higher affinity and selectivity, which resulted in greater bone density [94,95,96,97]. Parathyroid hormone-related protein (PTHrP) is a protein with homology to parathyroid hormone (PTH) at the amino terminus. Despite a common receptor (PTHR), PTH primarily acts as an endocrine regulator of calcium homeostasis, whereas PTHrP plays a fundamental paracrine role in the mediation of endochondral bone development. Abaloparatide is a peptide analog selected to maintain strong anabolic activity with decreased bone resorption, less calcium-mobilizing potential, and improved room temperature stability. Leder et al. have shown that 24 weeks of abaloparatide increases BMD of the spine and hip in a potentially clinically meaningful way. The abaloparatide-induced increases in lumbar spine BMD were robust and the BMD increases at the total hip were greater than with teriparatide [95].

Research by Makino et al. showed that the administration of abaloparatide to mice increased BMD more than teriparatide, as in humans when the frequency of administration was adjusted by the rate of bone turnover. Abaloparatide enhanced the anabolic window more than teriparatide, leading to bone gain, including trabecular and cortical bone. The mechanism by which abaloparatide shows a favorable balance of bone turnover may be partly due to enhanced remodeling-based bone formation [96]. Studies in estrogen-deficient ovariectomized rats indicate significant increases in bone formation, bone mass, and bone strength when abaloparatide is administered for several weeks to several months at doses as low as 0.25–1.0 μg/kg/d. Studies in rats and non-human primate showed no increases in biochemical or histomorphometric bone resorption parameters, with abaloparatide at the highest doses tested, which ranged up to 25 μg/kg [97].

## 3. New Trends in RANK/RANKL Signaling Inhibition—The Role of Meso and Antiprogestins—New SPRMs

Although the RANK/RANKL signaling pathway was first identified as a critical player in bone remodeling, the additional function of this pathway in the immune system control as well in hormone-dependent cancers development through its integration with sex hormones signaling into their physiological function has been recently discovered and researched. Considering the linkage between sex hormones and RANK/RANKL complex expression, it has been proven that progesterone enhanced RANKL expression in cells with high estrogen and progesterone receptors (ER/PR) activation [98,99], through the autocrine and paracrine mode (Figure 1). Activation of both of these modes allows the proliferation of not only PR+ but also PR- cells, which means that all types of breast cancer can develop with strong primary proliferative response of the mammary epithelium cells. Therefore, the RANK/RANKL axis seems to be an important mediator of progesterone-driven mammary epithelial cell proliferation, with a potential contribution to breast cancer initiation, progression, and metastasis [100]. Several studies have established the role of RANKL as a metastatic agent of cancer cells progression. Hence, it can be concluded that the RANKL/RANK axis is the fundamental molecular target for meso and antiprogestins with potential epithelial carcinogenesis inhibition [51].

It has been proven that the inhibitory effect of RANK-Fc on RANKL reduces the progression of breast cancer controlled by hormones and carcinogens [70]. Conversely, decreased level of OPG is associated with an increased incidence of breast cancer [101]. As a result of RANKL expression, in addition to mammary epithelial cell proliferation and breast cancer progression, the effect of the RANKL/RANK system on bone and lung metastases in mice according to the same pathway has been also observed. Accordingly, anti-RANKL treatment may be an interesting way to prevent and treat breast cancer and metastases [51,102,103].

New selective PR modulators (SPRMs) appear as potential anti-RANKL agents with benefits for patients with osteoporosis also occurring during breast cancer metastasis to bones. SPRMs represent the class of PR ligands that exert clinically significant tissue-selective progesterone action as its agonist, antagonist, or mixed agonist/antagonist on various progesterone-targeted tissues [104]. Action of SPRM as an antagonist, agonist, or mesoprogestin depends on its structure interaction with a PR receptor, leading to the activation or inactivation of a specific binding domains, which influences the associated co-repressor or co-activator of PR. SPRM’s activities vary with the type of tissue and the physiological context and may vary depending on the type of targeted cells [105].

**Figure 3:**
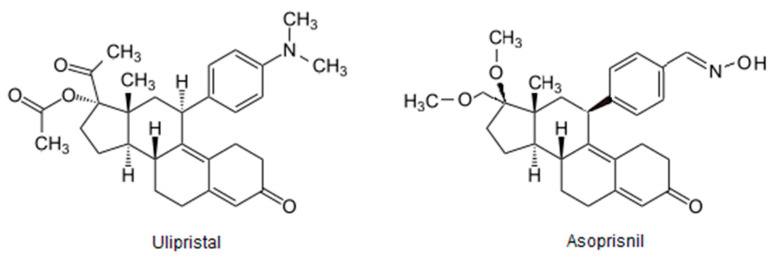
SPRM structures.

Modern SPRMs with anti- and mesoprogestin function similar to ulipristal and asoprisnil (Figure 3), and have been tested as potential anti-RANKL and anti-proliferation agents via NF-κB-cyclin D1 axis inhibition [106]. Tested in vitro new SPRMs showed the possibility to block RANK receptor activity via direct interaction. The destructive effect of tested SPRMs into the RANK/RANKL complex makes sense to recognize this class of SPRMs as potential anti-RANKL agents with a new perspective into osteoporosis treatment for postmenopausal women and for women during breast cancer therapy that occurs with loss of bone density.

## 4. Bone Homeostasis Evaluation—In Vivo and In Vitro Tests for Studying Osteoclastogenesis 

Bone homeostasis is orchestrated by osteoblasts and osteoclasts. This process of bone renovation and reconstruction are subjected for both mechanistic- and metabolic-regulated bone homeostasis, which is related to the regulation of calcium concentration in the plasma [11].

The in vitro and in vivo studies described in the literature have provided some evidence that inhibition of RANK/RANKL signaling pathway may result in bone homeostasis maintaining and tumor cells proliferation suppression. Based on these findings, we know that estrogen enhances OPG production and inhibits RANKL expression, whereas testosterone and nonaromatizable androgen 5a-DHT exert a reverse action. Disturbances in OPG concentrations appear during sex hormone deficiency may serve as a protective mechanism in men or a causative agent in postmenopausal osteoporosis period in women [107,108].

Since the RANK/RANKL pathway has been discovered and its engagement into osteoporosis has been proven, scientists search new active substances with antiosteoporosis activity with down-regulation of RANK/RANKL signaling among natural and synthetic compounds.

A polysaccharide (DAP), isolated from the roots of Dipsacus asper Wall, which includes galactose and mannose, has been tested in ovariectomized (OVX) rats. Administration of DAP significantly prevented OVX-induced bone loss in rats. The elicited mechanism of antiosteoporotic effect was mediated by the up-regulation of OPG and down-regulation of RANK and RANKL in both protein and mRNA expression in OVX rats. This shows that DAP can be clinically used as a potential alternative medicine for the prevention and treatment of postmenopausal osteoporosis [105,109,110].

A similar effect was obtained in in vivo studies conducted by Cai et al. with natural flavonoid taxifolin administered to ovariectomized mice. The tested substance reduced the level of RANKL expression and inhibited the activity of osteoclasts. Thus, taxifolin has been indicated as an inhibitor of osteoclastogenesis by the regulation of the RANK/RANKL signaling pathway and reduction of NF-κB activation. Taxifolin also prevented the production of reactive oxygen species (ROS) [111]. This is an important property because the dysregulation of ROS production affects the development of osteoporosis.

Synthetic triterpenoid (Nrf2 activator RTA-408) inhibits the production of reactive oxygen species and NF-κB signaling. As demonstrated by in vivo experiments, due to these properties, the compound prevents osteoclastogenesis in mice [112].

In vivo and in vitro studies indicate that L-tetrahydropalmatine (L-THP), an active alkaloid derived from corydalis, inhibited osteoclastic differentiation at an early stage, and also down-regulated the transcription level of osteoclastogenesis-related genes and impaired osteoclasts functions. The electrophoretic mobility shift assay (EMSA) revealed that the DNA binding activity of NF-κB was suppressed upon alkaloid treatment, and ultimately inhibits the expression of nuclear factor of activated T cells (NFATc1). The studies demonstrated that L-THP suppressed osteoclastogenesis in mice through RANK-TRAF6 interactions inhibition as well as an NF-κB pathway blockade. L-THP has been indicated to be a promising agent for treating osteoclastogenesis-related diseases such as post-menopausal osteoporosis [113,114,115,116,117].

Some papers also inform that genistein, an isoflavone isolated from soybean, may regulate cellular bone metabolism; therefore, this natural compound appears as a promising active substance to treat postmenopausal osteoporosis, although its mechanism of action stays still unrecognized [118,119].

Additionally, recently provided in vitro results obtained by Zhang et al. suggest that various breast cancer cell lines overexpress the RANK/RANKL signaling pathway, which was indicated by a higher level of RANK and RANKL proteins and their transcription expression in cancer cells. This research team showed that RANK and RANKL expression has been declined significantly under murrayanine treatment. This indicates that murrayanine targeted the RANK/RANKL pathway in breast cancer cells and inhibited their growth. Thus, it confirms that the repression of RANK induces apoptosis in cancer cells [120].

## 5. Conclusions

RANK-L, its receptor RANK, and the decoy receptor OPG are the key regulators for osteoclast development and the activation of mature osteoclasts. Bone metabolism is thus regulated by a balance between RANKL/RANK signaling and OPG level. The same molecules that regulate osteoclastogenesis are strongly associated with serum level changes of female sex hormones such as progesterone and estrogen. Reduced estrogen level, an inhibitor of bone resorption, contributes to the development of postmenopausal osteoporosis. In turn, progesterone increases the level of RANKL in PR+ expressed cells, mainly in natural mammary as well in breast cancer cells, and influences on the proliferation of epithelial cells. Then, as a result of biochemical links, the action of the RANK/RANKL pathway induces excessive bone resorption.

Currently, the medical treatment of osteoporosis is based on the use of the specific RANK inhibitors or drugs that can bind bone minerals that disrupt the formation of osteoclasts or initiate their apoptosis. Nevertheless, an increase of bone density without changes in flexibility or reduction of the bone remodeling rate may not lead to the desired functional improvement of organs. New drugs targeted the progesterone receptor (PR) present a potential future for osteoporosis treatment. Selective progesterone receptor modulators can induce a unique blend of PR-agonistic and -antagonistic changes, and lead to the inactivation of specific binding domains of PR and exhibit anti-RANKL and anti-proliferative effects.
